# Effect of an isolated active compound (Cg-1) of *Cassia glauca* leaf on blood glucose, lipid profile, and atherogenic index in diabetic rats

**DOI:** 10.4103/0253-7613.56076

**Published:** 2009-08

**Authors:** Papiya Mitra Mazumder, Mamta Farswan, V. Parcha

**Affiliations:** Birla Institute of Technology, Ranchi, Jharkhand, India; 1 Department of Pharmaceutical Sciences, SBS (PG) Institute of Biomedical Sciences, Balawala, Dehradun, Uttarakhand, India

**Keywords:** Blood glucose, *β*-sitosterol, *Cassia glauca*, diabetes, lipid profile, streptozotocin

## Abstract

**Objectives:**

The objective of present study was to evaluate the effect of active principle (Cg-1) from *Cassia glauca* leaf on serum glucose and lipid profile in normal and diabetic rats.

**Materials and Methods:**

Diabetes was induced by streptozotocin in neonates. Oral administration of petroleum ether, chloroform, acetone, and methanol of *C. glauca* leaf (100 mg/kg, p.o.) for 21 days caused a decrease in fasting blood glucose (FBG) in diabetic rats. Among all the extracts, acetone extract was found to lower the FBG level significantly in diabetic rats. Glibenclamide was used as standard antidiabetic drug (5 mg/kg, p.o). Acetone extract was subjected to column chromatography that led to isolation of an active principle, which was given trivial name Cg-1. Cg-1 (50 mg/kg, p.o.) was studied for its hypoglycemic and hypolipidemic potential. The unpaired *t*-test and analysis of variance (ANOVA) followed by post hoc test was used for statistical analysis.

**Results:**

Cg-1 caused a significant reduction in FBG level. It also caused reduction in cholesterol, triglycerides, and LDL levels and improvement in the atherogenic index and HDL level in diabetic rats.

**Conclusion:**

Improvement in the FBG and the atherogenic index by Cg-1 indicates that Cg-1 has cardioprotective potential along with antidiabetic activity and provides a scientific rationale for the use as an antidiabetic agent.

## Introduction

Diabetes mellitus is a disease characterized by disturbed metabolism and abnormally high blood sugar levels resulting from the body's inability to produce or properly use insulin.Instances of diabetes have increased and it is expected that there will be over 221 million people with diabetes worldwide by the year 2010.[1] Diabetes mellitus occurs in several forms. Approximately 10% of diabetes patients have type 1 diabetes mellitus, an autoimmune disease that destroys insulin-producing beta cells in the pancreas leading to a decrease in the concentration of insulin in the body, and the remainder have type 2 (non-insulin-dependent diabetes mellitus). Type 2 diabetes mellitus is a metabolic disorder characterized by a progressive decline in insulin action.[2] Beside these common types, other forms of diabetes are gestational diabetes, feline diabetes, and juvenile diabetes. The β cells normally compensate for insulin resistance by secreting greater amount of insulin in order to maintain glucose homeostasis. In non-insulin-dependent diabetes mellitus (type 2 DM) this β cell function becomes impaired due to insulin resistance leading to deterioration in glucose homeostasis and a subsequent development of impaired glucose tolerance (glucose resistance in the body which leads to high blood sugar level).[3,4] Hyperglycemia in diabetic patients is associated with alteration in glucose and lipid metabolism and modification in liver enzyme level.[5] Diabetes mellitus is recognized as a major risk factor for cardiovascular diseases (CVD) such as atherosclerosis, heart attack, stroke etc. About 75% of deaths among men with diabetes and 57% among women with diabetes are attributable to CVD.[6] Diabetes is associated with abnormalities of lipid metabolism and an increase in the atherogenic index.

Insulin and oral hypoglycemic agents are the most widely used drugs for lowering blood sugar in diabetes, but these drugs also have various side effects such as hypoglycemia, weight gain (sulfonylurea), and lactic acidosis (biguanides), and all of these drugs can causes liver and renal damage.[7]

More than 800 plants are used as traditional remedies for the treatment of diabetes throughout the world.[8] However, a scientific proof of the antidiabetic activity of medicinal plants and phytopharmaceuticals with fewer side effects is still lacking.

*C*assia glauca** Linn. (Caesalpiniaceae) is an evergreen shrub that grows about 10 ft high. It is widely distributed in the Uttarakhand state of India. Aerial parts of the plant are used as a central nervous system depressant, purgative, antimalarial, and as a diuretic.[9] The bark and leaves have been used in diabetes for lowering blood glucose level and gonorrhea in the Ayurvedic system of medicine.[9] *C. glauca* can easily survive in pollution and help to reduce chemical pollutants in the atmosphere.[10] Different species of *Cassia* such as *C. alata*[11] and C.*auriculata*[12] have also been studied and well documented for their antidiabetic potential.

The present study was planned to compare the blood glucose-lowering activity of different extracts/fractions of *C. glauca* in order to isolate the principle responsible for the antidiabetic activity of the plant. Further study was carried out to evaluate the antidiabetic and hypolipidemic effects of active principle (Cg-1) from *C. glauca* leaf extracts on normal and diabetic rats.

## Materials and Methods

### Preparation of different plant extracts

*C. glauca* leaves were collected from the forest of Dehradun in the month of April. The plant was identified and authenticated. Fresh plant leaves were shade dried at room temperature, ground into fine powder, and then extracted (amount 450 g) with solvents of increasing polarity such as petroleum ether, chloroform, acetone, and methanol, for 24 hours with each solvent, by hot extraction using the soxhlet apparatus at a temperature of 60°C. The extracts were concentrated under reduced pressure using a rotary evaporator to constant weight. The extracts were collected and preserved in a desiccator until used for further studies.

### Phytochemical study

A portion of residue from each extract was subjected to phytochemical analysis in order to see the presence of sterols, alkaloids, carbohydrates, tannins, phenols etc. in the leave extracts.[13,14]

### Isolation of active principle (Cg-1) from Cassia glauca leaf

All the four extracts of *C. glauca* leaf were screened for their effect on FBG level in diabetic rats. The acetone extract was found to show maximum reduction in blood glucose level; therefore, attempts were made to isolate the active principle from the acetone extract. The extract was subjected to column chromatography using silica gel mesh (200-400 size) as adsorbent and CHCl_3_: MeOH in different ratio mobile phase which led to isolation of some compounds based on thin layer chromatography (SiO_2_ and CHCl_3_: MeOH). All of these compounds were screened for their effect on FBG level. Among the isolated compounds, one compound which was given trivial name Cg-1 showed maximum reduction in FBG level and was used for further study.

### Acute toxicity studies

Acute toxicity studies were carried out on Swiss albino mice.[15] The active acetone extract at doses of 100, 300, 500, 1000 and 3000 mg/kg was administered to five groups of mice, each group containing six animals. After administration of extracts, the animals were observed for the first 3 hours for any toxic symptoms followed by observation at regular intervals for 24 hours up to 7 days. At the end of study, the animals were also observed for general organ toxicity, morphological behavior and mortality.

### Pharmacological activity

#### Animals

Wistar albino rats of either sex (of age 6 months and weighing 350 g) were bred in the Institutional animal house. The animals were housed in standard polypropylene cages and maintained under controlled room temperature (22 ° 2±C) and humidity (55 ° 5%) with 12:12 hour light and dark cycle. All the animals were provided with commercially available rat normal pellet diet and water *ad libitum.* The guidelines of the Committee for the Purpose of Control and Supervision of Experiments on Animals (CPCSEA) of the Government of India were followed and prior permission was granted from the Institutional Animal Ethics Committee (No. 273/CPCSEA).

### Induction of diabetes

The method of Portha *et al.*[16] was followed for the induction of diabetes. Diabetes mellitus was induced in 5-day-old neonates (50 animals) by an intraperitonial injection of streptozotocin (90 mg/kg in 0.1M citrate buffer pH 4.5). The control group received equivalent amount of citrate buffer. The pups were allowed to live with their respective mothers and weaned from breastfeeding at 4 weeks of age. Eight weeks after injection of streptozotocin, blood sample was taken from the animals, serum was separated out, and fasting blood glucose (FBG) level was checked by the glucose oxidase-peroxidase method using Span Diagnostic kits. Animals showing FBG more than 150 mg/dl were considered as diabetic (38 animals) and included for the study.

### Treatment protocol

The diabetic animals were divided into six groups each containing six animals, and one group of normal non-diabetic animals. All the four extracts of *C. glauca* leaf were given at a dose of 100 mg/kg, orally daily for a period of 21 days as a suspension in Tween 80 to different groups of diabetic animals.

Group I : Normal animals received Tween 80 in a dose of 1% suspension in distilled water.

Group II : Diabetic animals received Tween 80 in a dose of 1% suspension in distilled water.

Group III : Diabetic animals received standard antidiabetic drug glibenclamide (5 mg/kg, p.o.)

Group IV : Diabetic animals received petroleum ether extract (PEE, 100 mg/kg, p.o.)

Group V : Diabetic animals received chloroform extract (CE, 100 mg/kg, p.o)

Group VI : Diabetic animals received acetone extract (AE, 100 mg/ kg, p.o)

Group VII : Diabetic animals received methanol extract (ME, 100 mg/kg, p.o.)

At the end of the experimental period, the animals were fasted overnight for 8 hours and a blood sample was taken from the retro-orbital plexus under mild ether anesthesia, serum was separated out, and FBG level was measured by the glucose oxidase-peroxides method using Span Diagnostic kits.

After the pharmacological screening, it was found that the acetone extract showed a maximum decrease in FBG level. It was subjected to column chromatography and active principle (Cg-1) was isolated from the acetone extract.

### Effect of Cg-1 on FBG, lipid profile, and the atherogenic index in diabetic rats

The diabetic animals were divided into three groups each containing six animals, and one group of normal non-diabetic animals. The animals received following treatment daily for a period of 21 days.

Group I : Normal animals received Tween 80 in a dose of 1% suspension in distilled water.

Group II : Diabetic animals received Tween 80 in a dose of 1% suspension in distilled water.

Group III : Diabetic animals received standard antidiabetic drug glibenclamide (5 mg/kg, p.o.).

Group IV : Diabetic animals received Cg-1 (50 mg/kg, p.o.).

At the end of the experimental period, the animals were fasted overnight for 8 hours and a blood sample was taken from the retro orbital plexus under mild ether anesthesia, serum was separated out and FBG level was measured by the method of glucose oxidase-peroxides using Span Diagnostic kits.

Serum was used to measure the lipid profile of diabetic rats. Cholesterol level was determined by the enzymatic method,[17] triglyceride by the enzymatic colorimetric method[18] and HDL and LDL by the phosphotungustate method[19] using span diagnostic kits. The atherogenic index was calculated by using the following formula.

$\text{Atherogenic index}=\frac{\text{Total serum cholesterol}}{\text{Total HDL}-\text{cholesterol}}$

### Drugs and chemicals

Streptozotocin was purchased from Calbiochem, Germany. Standard antidiabetic drug glibenclamide was obtained from Ranbaxy Research Laboratories, Gurgaon, India. Analytical grade chemicals, including various organic solvents (petroleum ether, chloroform, acetone, and methanol) from E. Merck India Ltd and Ranbaxy laboratories India, were used for the extraction and the phytochemical study of the constituents.

### Statistical analysis

The results were expressed as mean ± SEM. The unpaired *t*-test was used for analyzing the data between two groups. Statistical analysis of data among the groups was performed by using analysis of variance (ANOVA) followed by the Tukey test of significance.

## Results

### Phytochemical study

After phytochemical investigation, it was found that PEE of the leaf showed the presence of sterols. CE showed the presence of carbohydrates and alkaloids. AE and ME showed the presence of alkaloids and tannins. These tests were performed to see the chemical nature of the extracts. Since Cg-1 was found to be the active principle of *C. glauca* leaf, preliminary tests were carried out to establish its structure. Cg-1 has a melting point of 105°C. We found that Cg-1 is a polyphenolic compound. Further study of detailed spectral analysis is needed to establish the complete structure of Cg-1 by which we can find a lead structure on which further work could be carried out.

### Acute toxicity studies

Acute toxicity studies revealed that *C. glauca* extracts did not produce any toxic symptoms when administered orally to mice. The lethal dose (LD50 value) was of 3 g/kg body weight.

### Effect of different leaf extracts of C. glauca on FBS of diabetic rats

All the extracts produced significant reduction in FBG level in diabetic rats [Table 1]. However, AE produced highest significant reduction (52%) in FBS as compared to that produced by other extracts [Table 1]. Glibenclamide produced significant reduction in FBG level (67%) which was highest as compared to that produced by all the extracts [Table 1].

**Table 1:** Effect of different *Cassia glauca* leaf extracts on fasting blood glucose of diabetic rats

*Different groups and treatment (p.o.)*	*Fasting blood glucose (mg/dl) (mean ±SEM)*		
	
	*Before treatment*	*After treatment*	*% reduction*
Normal (healthy animals) + Tween 80 (1 ml/kg)	90 ± 0.8	96 ± 1.1	-
Diabetic control + Tween 80 (1 ml/kg)	241 ± 1.3^a^	235 ± 0.8^a^	2
Diabetic + glibenclamide (5 mg/kg)	260 ± 1.1	90 ± 1.3***	67
Diabetic + PEE (100 mg/kg)	229 ± 1.3	130 ± 1.1**	35
Diabetic + CE (100 mg/kg)	236 ± 1.7	134 ± 1.3**	43
Diabetic + AE (100 mg/kg)	224 ± 2.6	107 ± 1.4***	52
Diabetic + ME (100 mg/kg)	220 ± 3.8	129 ± 1.5**	41

** *P* < 0.01;

*** *P* < 0.001 as compared to their corresponding value before treatment.

a *P* < 0.001 as compared to their corresponding value in normal group. (n=6 in each group)

### Effect of Cg-1 on FBG and the Lipid Profile of Diabetic Rats

Cg-1 produced 63% reduction in FBG where as glibenclamide caused 67% reduction in FBG in diabetic rats [Table 2]. Cg-1 also caused significant (*P* < 0.01) reduction in the levels of cholesterol, triglyceride, and LDL, whereas glibenclamide caused highest significant (*P* < 0.001) reduction in the levels of cholesterol, triglyceride, and LDL [Table 3]. Cg-1 as well as glibenclamide caused significant improvement in the level of HDL-cholesterol in diabetic animals [Table 3].

**Table 2:** Effect of Cg-1 on fasting blood glucose of diabetic rats

*Groups and treatment (p.o.)*	*Fasting blood glucose (mg/dl) (mean ±SEM)*		
	
	*Before treatment*	*After treatment*	*% reduction*
Normal (healthy animals) + Tween 80 (1 ml/kg)	94 ± 0.8	97 ± 1.1	-
Diabetic control + Tween 80 (1 ml/kg)	245 ± 1.3^a^	232 ± 0.8^a^	5
Diabetic + glibenclamide (5 mg/kg)	260 ± 1.1	90 ± 1.3*	67
Diabetic + Cg-1 (50 mg/kg)	249 ± 0.8	93 ± 0.8*	63

* *P* < 0.001 as compared to their corresponding value before treatment.

a *P* < 0.001 as compared to their corresponding value in normal group. (n=6 in each group)

**Table 3:** Effects of Cg-1 on lipid profile of diabetic rats

*Groups and treatment (p.o.)*	*Values (mg/dl) (mean ± SEM)*			
	
	*Cholesterol*	*HDL*	*LDL*	*Triglyceride*
Normal (healthy animals) + Tween 80 (1 ml/kg)	98 ± 0.9	36 ± 1.2	52 ± 1.0	50 ± 2.5
Diabetic control + Tween 80 (1 ml/kg)	166 ± 3^a^	28 ± 0.9^a^	108 ± 0.4^a^	149 ± 0.9^a^
Diabetic + glibenclamide (5 mg /kg)	102 ± 1.6***	34.8 ± 1.2***	54.8 ± 0.62***	70 ± 0.45**
Diabetic + Cg-1 (50 mg/kg)	120 ± 1.5**	31 ± 2.2***	63 ± 2.1**	90 ± 0.4*

* *P* < 0.05,

** *P* < 0.01,

*** *P* < 0.001 as compared to their corresponding value in diabetic control group.

a *P* < 0.001 as compared to their corresponding value in normal group. (n = 6 in each group)

In diabetic control group, protection was 0% and in normal healthy animals it was 61.5% [Table 4]. Both glibenclamide and Cg-1 produced a marked decrease in the atherogenic index in diabetic animals. Cg-1 caused 49% protection, whereas glibenclamide caused 56% protection in diabetic rats.

**Table 4:** Effect of Cg-1 on the atherogenic index of diabetic rats

*Group and treatment (p.o.)*	*Atherogenic index*	Protection* (%)
Normal (healthy animals) + Tween 80 (1 ml/kg)	1.5	61.5
Diabetic control + Tween 80 (1 ml/kg)	3.9	0
Diabetic + glibenclamide (5 mg/kg)	1.7	56
Diabetic + Cg-1 (50 mg/kg)	2.0	49

n = 6 in each group		
${\text{Protection}}^{*}\left(\%\right)=\frac{\text{Atherogenic index of diabetic control}–\text{Atherogenic index of treated group}}{\text{Atherogenic index of diabetic control group}}\times 100$		

## Discussion

Administration of streptozotocin caused rapid destruction of pancreatic β cells in rats, which led to impaired glucose-stimulated insulin release and insulin resistance, both of which are marked feature of type II diabetes.[20] The blood glucose-lowering effect of plant extracts is generally dependent upon the degree of pancreatic β-cell destruction and useful in moderate streptozotocin-induced diabetes.[21] The lesser the degree of pancreatic β-cell destruction, the more useful the herb is in treating diabetes in animals.

In general, an increase in blood glucose level is usually accompanied by an increase in plasma cholesterol, triglyceride, LDL levels and a decrease in HDL levels as observed in diabetic patients.[22] The marked hyperlipidemia (increase in the level of lipid in the body) that characterizes the diabetic state may be the consequence of the uninhibited actions of lipolytic hormones on fat depots.[23] Among all the extracts tested, the acetone extract produced significant reduction in the blood glucose level comparable to that produced by glibenclamide treatment. Further acetone extract was purified by column chromatography that led to isolation of a several specific compounds. Of the isolated compounds, Cg-1 possesses significant blood glucose-lowering and cholesterol-lowering activities. The improvements in the lipid profile in diabetic animals after treatment with Cg-1 could be beneficial in preventing diabetic complications, as well as improving lipid metabolism in the kidneys of diabetic patients.[24]

Phytochemical investigation of Cg-1 showed that it is a polyphenolic compound. Previous research on this subject has shown that drugs containing tannins and sterols possess antidiabetic activity.[25] Based on this evidence, Cg-1 can be regarded as the principle responsible for the antidiabetic effect of *C. glauca* leaf.

Cg-1 from the acetone extract of *C. glauca* leaf has shown significant reduction in blood glucose and alteration in the lipid profile in diabetic rats. At present, the exact mechanism for the above action is not known and further detail work is required to ascertain the same.

A decrease in the FBG level, improvement in the lipid profile along with the decrease in the atherogenic index by Cg-1 suggests that Cg-1 could be useful as an antidiabetic agent with cardio-protective activity. Further study is required to derived the complete structure of Cg-1 which will be a lead compound, on which structure-activity relationship would be carried out, which could be useful as an alternative cure to oral hypoglycemics, in the management of diabetes mellitus.
